# *IRLnc*: a novel functional noncoding RNA contributes to intramuscular fat deposition

**DOI:** 10.1186/s12864-020-07349-5

**Published:** 2021-02-01

**Authors:** Ligang Wang, Zhong-Yin Zhou, Tian Zhang, Longchao Zhang, Xinhua Hou, Hua Yan, Lixian Wang

**Affiliations:** 1 Key Laboratory of Farm Animal Genetic Resources and Germplasm Innovation of Ministry of Agriculture of China, Institute of Animal Sciences, Chinese Academy of Agricultural Sciences, Beijing, 100193 China; 2 State Key Laboratory of Genetic Resources and Evolution, Kunming Institute of Zoology, Chinese Academy of Sciences, Kunming, 650223 China; 3 State Key Laboratory Breeding Base of Dao-di Herbs, China Academy of Chinese Medical Sciences National Resource Center for Chinese Materia Medica, Beijing, 100021 China

**Keywords:** Intramuscular fat, Long non-coding RNA, Insulin resistance, Pig

## Abstract

**Background:**

Intramuscular fat (IMF) is associated with meat quality and insulin resistance in animals. Research on genetic mechanism of IMF decomposition has positive meaning to pork quality and diseases such as obesity and type 2 diabetes treatment. In this study, an IMF trait segregation population was used to perform RNA sequencing and to analyze the joint or independent effects of genes and long intergenic non-coding RNAs (lincRNAs) on IMF.

**Results:**

A total of 26 genes including six lincRNA genes show significantly different expression between high- and low-IMF pigs. Interesting, one lincRNA gene, named IMF related lincRNA (*IRLnc*) not only has a 292-bp conserved region in 100 vertebrates but also has conserved up and down stream genes (< 10 kb) in pig and humans. Real-time quantitative polymerase chain reaction (RT-qPCR) validation study indicated that nuclear receptor subfamily 4 group A member 3 (*NR4A3*) which located at the downstream of *IRLnc* has similar expression pattern with *IRLnc*. RNAi-mediated loss of function screens identified that *IRLnc* silencing could inhibit both of the RNA and protein expression of *NR4A3*. And the in-situ hybridization co-expression experiment indicates that *IRLnc* may directly binding to *NR4A3*. As the *NR4A3* could regulate the catecholamine catabolism, which could affect insulin sensitivity, we inferred that *IRLnc* influence IMF decomposition by regulating the expression of *NR4A3*.

**Conclusions:**

In conclusion, a novel functional noncoding variation named *IRLnc* has been found contribute to IMF by regulating the expression of *NR4A3*. These findings suggest novel mechanistic approach for treatment of insulin resistance in human beings and meat quality improvement in animal.

**Supplementary Information:**

The online version contains supplementary material available at 10.1186/s12864-020-07349-5.

## Background

Intramuscular fat (IMF) refers to the amount of fat located in skeletal muscle fibers [1]. Excess accumulation of IMF has been reported to be associated with diseases, such as type 2 diabetes and insulin resistance in humans [2]. In animals, as an important determinant of meat quality, IMF content directly influences flavor and juiciness and indirectly influences tenderness and meat color [1]. Moreover, pork IMF contains more unsaturated fatty acids (∼10–15% of total fatty acids) than beef and lamb [3]. Long-chain polyunsaturated fatty acids (LC-PUFA) such as omega-3 PUFA, eicosapentaenoic (EPA, 20:5n-3), and docosahexaenoic (DHA, 22:6n-3) acids are well accepted having beneficial effects on human brain development and cardiovascular disease [4, 5]. Both extremely high and extremely low IMF content is undesirable in consumed meat [1]. Thus, IMF is an important factor for human health.

It is generally accepted that IMF is a complex trait that is influenced by multiple genes or quantitative trait loci (QTLs). To date, a total of 709 QTLs have been reported to be associated with pig IMF content (PigQTLdb, http://www.animalgenome.org/cgi-bin/QTLdb/SS/index, released at April 26, 2020) [6]. However, the locations of these QTLs are not accurate due to the limited density of microsatellite markers. Long-term fine-mapping experiments are needed to refine these loci and investigate causative variants [7]. Furthermore, most of the single nucleotide polymorphisms (SNPs) associated with IMF in genome-wide association studies only explain a small part of the total genetic variance. Studies identifying genetic variation that explains this “missing heritability” of IMF are urgently needed [8].

Since they reside in regulatory elements of the genome, noncoding genomic variants located in intronic regions of protein-coding genes or in intergenic regions may have functional roles in the expression of specific phenotypes or traits. In pigs, long intergenic non-coding RNAs (lincRNAs) have been reported to be associated with permanent molars, adipose and muscle development, and energy metabolism [9–11]. Although several lincRNAs have been reported associated with pork and poultry IMF [12–15], little is known about the mechanism of lincRNA gene regulation in pig IMF.. The objectives of this work were to perform RNA sequencing analysis using an IMF character segregation population which construct using high IMF pigs (Min pig) and low IMF pigs (Large white pigs) crossbred and to analyze the joint or independent effects of lincRNAs on IMF. Moreover, we aimed to identify genetic markers that may be suitable for inclusion in animal genetic improvement programs and provide new targets for the treatment of insulin resistance in humans.

## Results

### RNA sequencing, data mapping, and transcript identification

RNA sequencing of longissimus dorsi muscle tissue has been done first in our research. After filtering, a total of 579.53 million clean reads (97.22% of the raw data) were obtained. More than 75% of the clean data could be mapped to the reference genome (v11.1 ftp://ftp.ensembl.org/pub/release-102/fasta/sus_scrofa/dna/). A total of 26,276 transcript units were identified, including 4671 lincRNAs. Among these 26,276 units, 59.7% encoded proteins, 3.4% were miscellaneous RNAs, 2.2% were microRNAs (miRNAs), 0.6% were mitochondrial ribosomal RNAs (rRNAs), 0.2% were small nuclear RNAs (snRNAs), and the remaining 33.6% were pseudogenes and processed transcripts. The clean data have been submitted to the Genome Sequence Archive, with the accession number CRA001645.

### Differentially expressed genes between high- and low-IMF content pigs

**Fig. 1:**
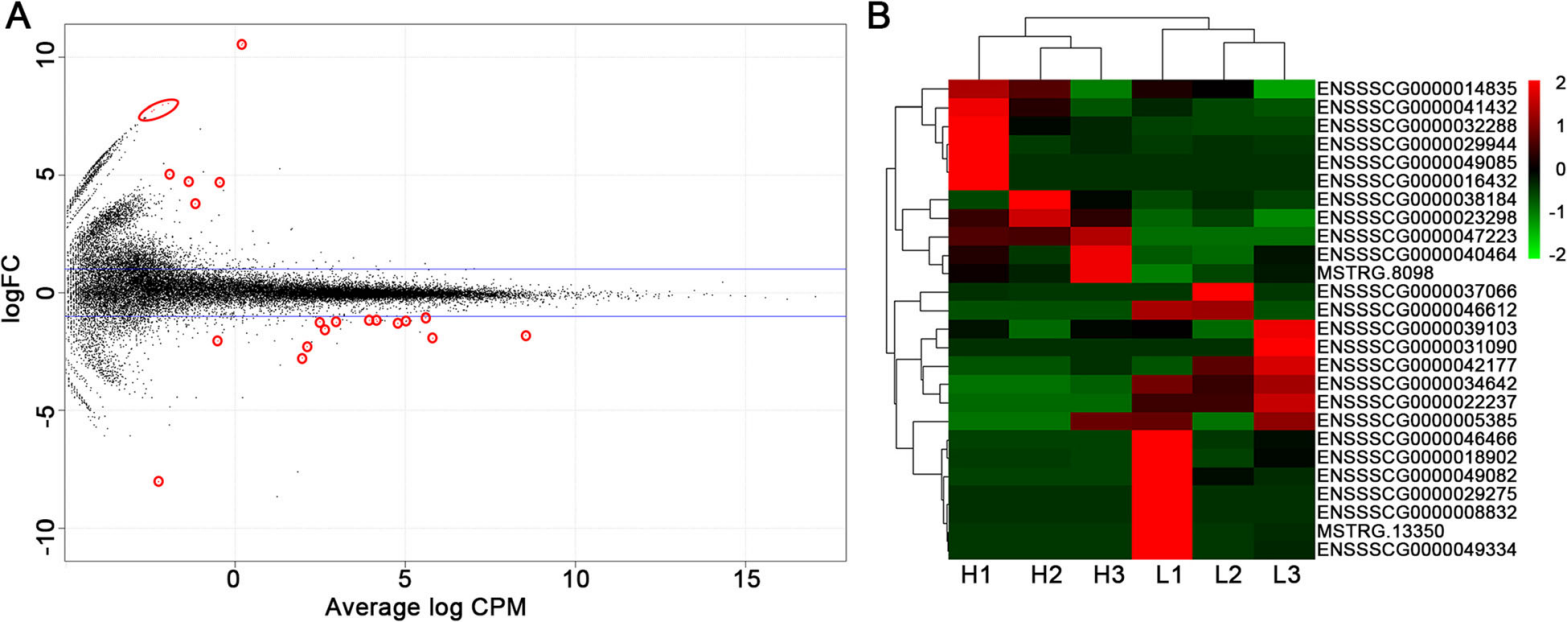
Significantly DE transcripts between high- and low-IMF pigs. *N*=3 in each group. **a** Volcano plot of significantly DE transcripts, the significant DE transcripts (FDR < 0.05) were in the color of red, blue lines indicate the threshold of log2 (0.67) and log2 (1.5). **b** Heat map and cluster represent the 26 DE transcripts differentially regulated in high- and low-IMF pigs, the color of scale bars represent the RPKM of each transcript

A total of 26 transcripts which have significantly differentially expression (DE,false discovery rate (FDR) < 0.1) between pigs with high and low IMF content were identified using a paired samples model in edgeR [16]. This included 5 novel protein-coding genes, 15 known protein-coding genes and 6 lincRNAs (Table 1 and Fig. 1). Six of the 20 protein-coding genes were upregulated with more than 2-fold changes (FC) in the pigs with low IMF content. And these six genes were N-Acetyl-Alpha-Glucosaminidase (*NAGLU*), novel gene 1 (*Novel*1), mitochondrially encoded NADH: ubiquinone oxidoreductase core subunit 6 (*ND6*), sushi domain containing 3 (*SUSD3*), novel gene 4 (*Novel*4), and leucine rich repeat containing 66 (*LRRC66*). Moreover, two lincRNAs were upregulated in the pigs with high IMF content.

**Table 1:** Description of significantly DE transcripts between IMF-differential pigs

Gene symbol	logFC	logCPM	*P*Value	FDR	Chromosome
*NAGLU*	10.55	0.22	2.48E-31	7.73E-27	12
*Novel1*	10.49	0.18	2.56E-18	4.00E-14	AEMK02000407.1
*ACBD7*	−2.78	1.97	5.52E-13	5.75E-09	10
*PPARGC1*	−1.80	8.55	8.39E-11	6.55E-07	8
*IRLnc*	−2.29	2.11	8.48E-09	5.30E-05	1
*Novel2*	−8.04	−2.25	2.99E-08	1.56E-04	18
*GADD45A*	−1.12	4.15	5.62E-07	2.48E-03	6
*IRLnc2*	7.95	−2.12	6.35E-07	2.48E-03	9
*IRLnc3*	7.68	−2.44	2.13E-06	7.40E-03	2
*NR4A3*	−1.94	5.82	2.44E-06	7.62E-03	1
*SRXN1*	−1.23	2.50	2.69E-06	7.64E-03	17
*IRLnc4*	−1.26	2.98	3.15E-06	8.21E-03	10
*LEP*	−2.05	−0.51	4.85E-06	1.16E-02	18
*SLC20A1*	−1.06	5.60	5.38E-06	1.16E-02	3
*FASN*	−1.21	4.18	5.57E-06	1.16E-02	12
*PRKAG2*	−1.31	4.79	6.90E-06	1.35E-02	18
*ND6*	4.67	−0.44	1.09E-05	1.89E-02	MT
*Novel3*	5.01	−1.94	1.09E-05	1.89E-02	AEMK02000635.1
*IRLnc5*	7.75	−2.38	1.19E-05	1.95E-02	17
*IRLnc6*	4.73	−1.37	1.38E-05	2.11E-02	11
*ADIPOQ*	−1.19	5.05	1.42E-05	2.11E-02	13
*SUSD3*	7.44	−2.68	1.57E-05	2.23E-02	3
*Novel4*	3.77	−1.18	2.58E-05	3.50E-02	AEMK02000297.1
*C2CD3*	−1.18	3.94	2.73E-05	3.55E-02	9
*Novel5*	−1.52	2.65	3.57E-05	4.35E-02	3
*LRRC66*	8.01	−1.96	3.62E-05	4.35E-02	8

*DE* differential expression. *IMF* intramuscular fat. *Gene symbol* names of the genes. *FC* fold change (low - IMF vs. high - IMF). *CPM* counts per million. *FDR* false discovery rate. *NAGLU* N-Acetyl-Alpha-Glucosaminidase, *Novel* novel gene, *ACBD7* Acyl-CoA Binding Domain Containing 7, *PPARGC1* Peroxisome proliferator-activated receptor-gamma coactivator-1, *IRLnc* IMF-related LincRNA, *GADD45A* Growth Arrest And DNA Damage-Inducible Protein GADD45 Alpha, *NR4A3* Nuclear receptor subfamily 4 group A member 3, *SRXN1* Sulfiredoxin 1, *LEP* Leptin, *SLC20A1* Solute Carrier Family 2 Member 1, *FASN* Fatty acid synthase, *PRKAG2* Protein Kinase AMP-Activated Non-Catalytic Subunit Gamma 2, *ND6* Mitochondrially Encoded NADH: Ubiquinone Oxidoreductase Core Subunit 6, *ADIPOQ* Adiponectin, *SUSD3* Sushi Domain Containing 3, *C2CD3* C2 Calcium Dependent Domain Containing 3, *LRRC66* Leucine Rich Repeat Containing 66

Among the 26 DE transcripts, IMF-related LincRNA (*IRLnc*) and nuclear receptor subfamily 4 group A member 3 (*NR4A3*), which had similar expression patterns, were located on chromosome 1. Solute carrier family 2 member 1 (*SLC20A1*), *SUSD3* and a novel transcript were located on chromosome 3. Peroxisome proliferator-activated receptor-gamma coactivator-1 (*PPARGC1*) and *LRRC66* were located on chromosome 8. IMF-related LincRNA2 and C2 calcium dependent domain Containing 3 (*C2CD3*), were located on chromosome 9. Acyl-CoA binding domain containing 7 (*ACBD7*) and IMF-related LincRNA4 were located on chromosome 10. *NAGLU* and fatty acid synthase (*FASN*) were located on chromosome 12. Sulfiredoxin 1 (*SRXN1*) and IMF-related LincRNA5 were located on chromosome 17. Leptin (*LEP*) and protein kinase AMP-Activated non-catalytic subunit gamma 2 (*PRKAG2*) were located on chromosome 18. Other DE transcripts were located on chromosome 2, 6, Mitochondrial and unmapped sequences.

### RT-qPCR validation of DE genes

**Fig. 2:**
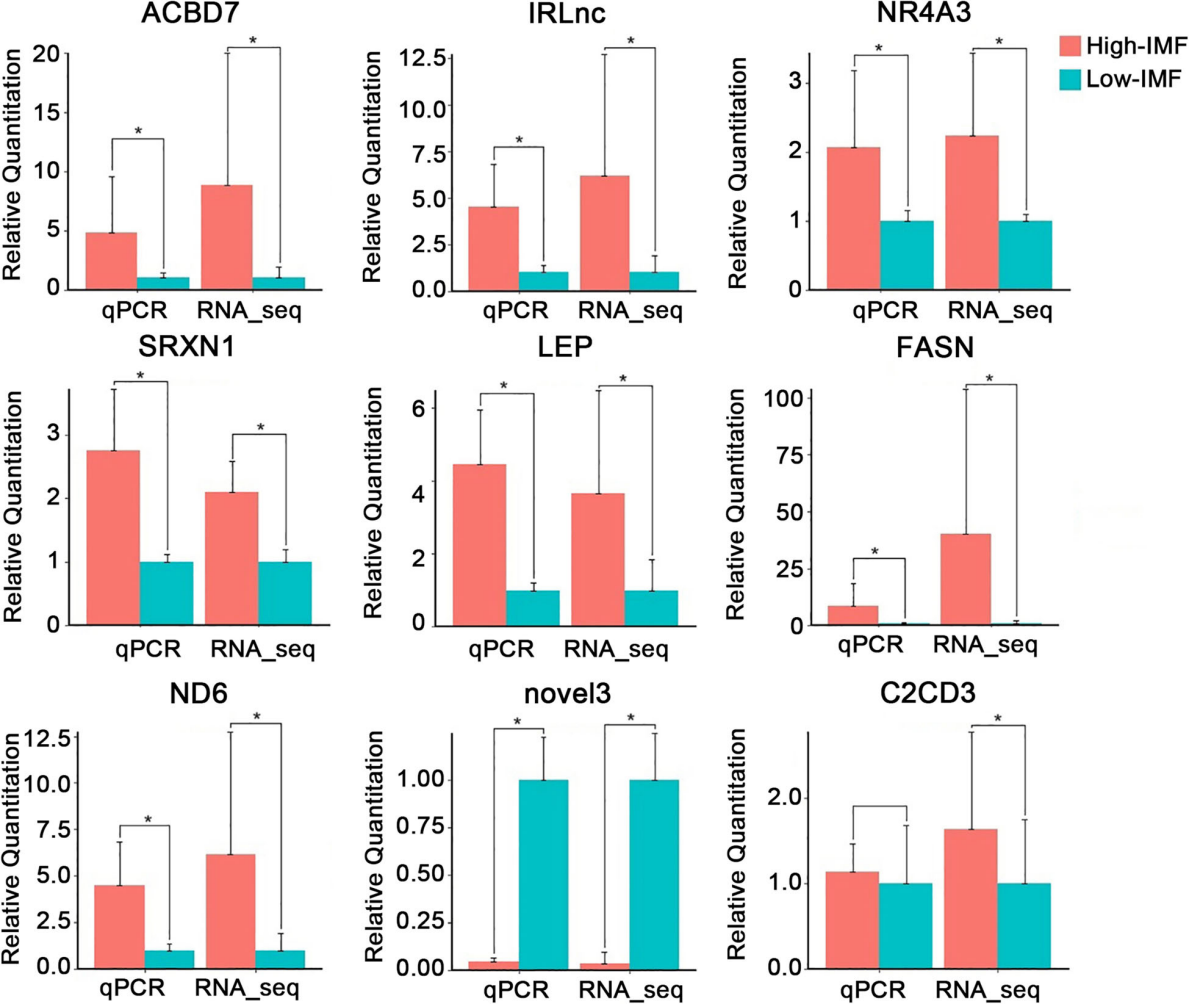
Validation of differentially expressed genes in high - and low - IMF pigs by RT-qPCR. For each gene, the value of qPCR and sequencing in low IMF group were set at 1. *N*=3 in each group, * represents *P*< 0.05. ACBD7: Acyl-CoA Binding Domain Containing 7, IRLnc: IMF-related LincRNA, NR4A3: nuclear receptor subfamily 4 group A member 3, SRXN1: Sulfiredoxin 1, LEP: Leptin, FASN: fatty acid synthase, ND6: NADH dehydrogenase subunit 6, novel3: novel protein coding gene 3, C2CD3: C2 Calcium Dependent Domain Containing 3

The same pigs with low and high IMF content in RNA-seq analysis were selected for validation by RT-qPCR. According to the RNA-seq abundance, we select 9 DE transcripts for RT-qPCR analysis. As relative quantitation of each transcript between the RT-qPCR and RNA-seq were not in same level, we set the value of qPCR and sequencing in low IMF group to be one. RT-qPCR results showed that 88.89% (8 of 9) of the selected transcripts could be validated in low IMF content vs. high IMF content pigs (Fig. 2). Among the eight validated DE transcripts, there are six known genes which were *ACBD7*, *NR4A3*, *SRXN1*, *LEP*, *FASN*, and *ND6*. One novel gene (*novel3*) and one lincRNA (*IRLnc*) could also been validated. Therefore, the sequencing results were reliable and candidate DE mRNAs and lincRNAs could be used for further analysis.

### Identification of sequence homology with 99 vertebrates and query of upstream and downstream genes of *IRLnc*

**Fig. 3:**
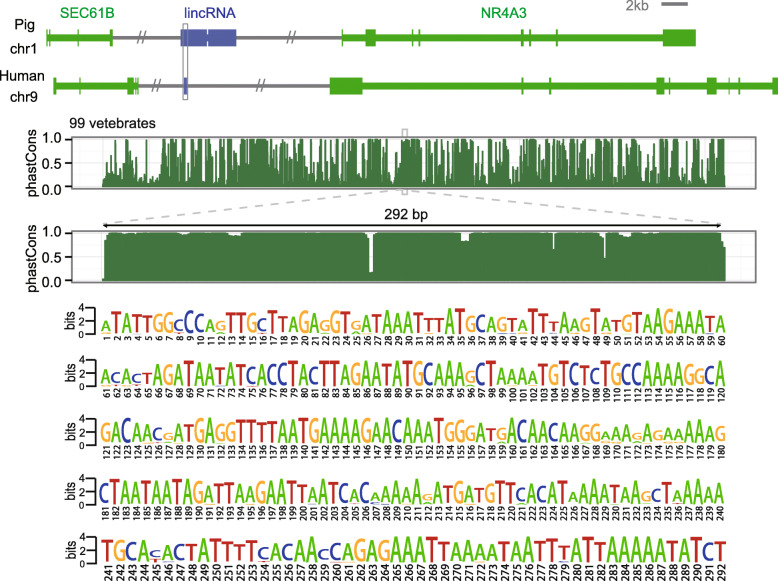
Synteny and sequence conservation of *IRLnc* PhastCon plot of pig and 99 vertebrates. The grey box shows the region with sequence conservation. The PhastCon plot is relative to loci in the human genome and is derived from 99 vertebrate whole-genome alignments. The consensus logo highlights the 292-bp conserved sequence, which was identified from the 99 vertebrate genome alignments. A score of 4 bits indicates that these bases are perfectly conserved in the 99 vertebrate genomes

Conservation analysis of 100 vertebrate whole genomes showed that there was a 292-bp region within the *IRLnc* gene (11199-bp) that was conserved between pigs and humans (Fig. 3). To determine whether *IRLnc* interacts with neighboring genes, we performed a sequence query analysis of the gene in the 500-kb window surrounding *IRLnc*. Two genes, Sec61 translocon beta subunit (*Sec61B*) and *NR4A3*, were found adjacent to *IRLnc*. We then analyzed the read counts and logFC values of *Sec61B* and *NR4A3* in RNA-seq data and found that *Sec61B* and *NR4A3* had logFC values of 0.03 and 1.94 in pigs with high IMF content compared to those with low IMF content, respectively (FDR = 1 and 0.0.0076, respectively), and this means no expression difference of *Sec61B* and significant expression differences of *NR4A3*.

### Gene expression pattern of *Sec61B* and *NR4A3* in low- and high- IMF pigs

**Fig. 4:**
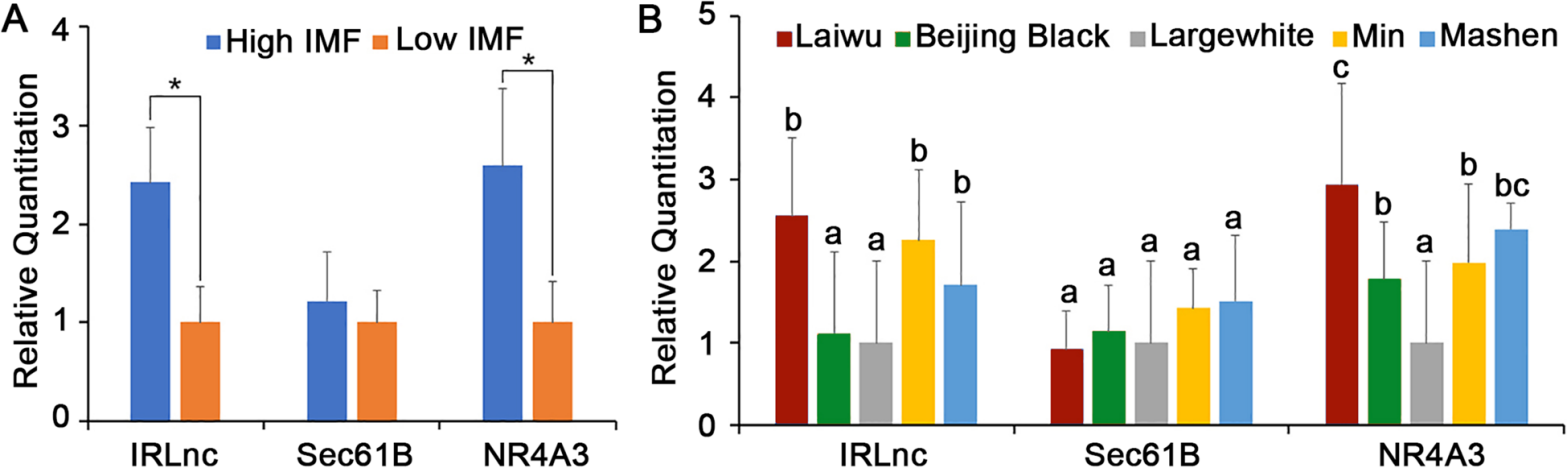
Gene expression of *IRLnc*, *Sec61B* and *NR4A3*. **a** The expression of *IRLnc, Sec61B* and *NR4A3* between high- and low-IMF pigs (*n*=5 in each group), * represents *P*< 0.05. **b** The expression of *IRLnc, Sec61B* and *NR4A3* in different breeds (*n*=5 in each breed), different characters above the bar represent significant differences. Laiwu is a pig breed with extremely high- IMF, Min and Mashen pigs have high IMF, Beijing Black pigs have median IMF, and Large White pigs have median to low IMF

To confirm the differential expression of *Sec61B* and *NR4A3*, we validated these findings in a bigger population (five pigs with low IMF content and five with high IMF content). As shown in Fig. 4a, there was no difference in *Sec61B* expression between the two groups. However, *NR4A3* gene expression was significantly different. The expression of *Sec61B* and *NR4A3* in five breeds pigs with different average IMF were also detected to infer the expression of *IRLnc* and its upstream and downstream genes (Fig. 4b). The results indicated that, the *NR4A3* gene expression was significantly high in Laiwu, Mashen, Min and Beijing Black pigs rather than in Large white (*P*< 0.05). And there are almost none differences of *Sec61B* expression between these pigs. Thus, we chose *NR4A3* for further research.

### LincRNA-RNA interaction prediction

**Fig. 5:**
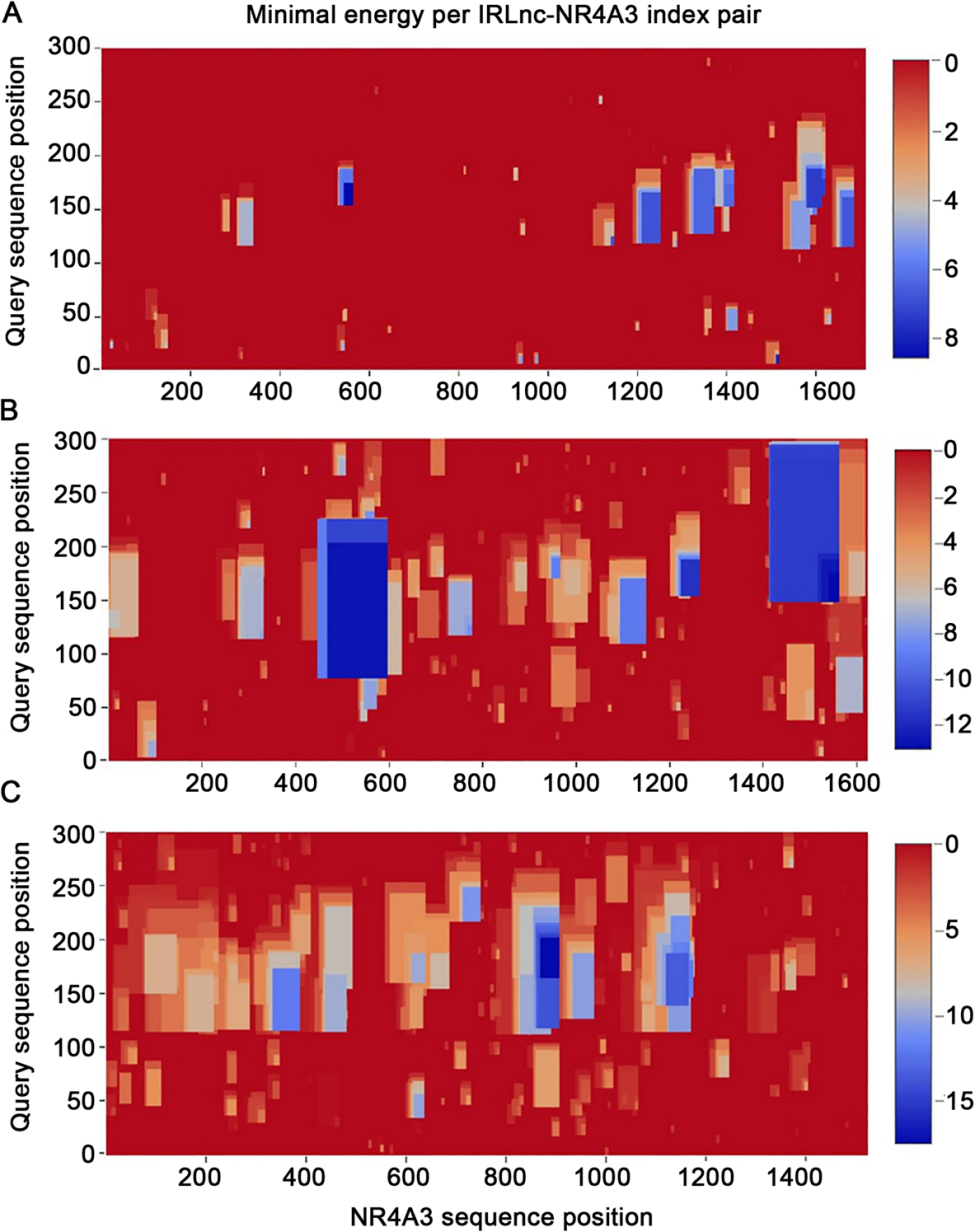
Minimal energy per IRLnc-NR4A3 index pair. **a-c** The minimal energy of three *NR4A3* mRNA segments to IRLnc index pairs. The color of scale bar indicates the binding energy of each domain of the sequence. Y-axis is the relative position of the three segments

In order to explore the potential lincRNA-RNA interaction, we calculate the interaction energy of *IRLnc* and *NR4A3*. Since IntaRNA software could only analyze sequences less than 2000 bp, we divided the *NR4A3* mRNA sequence into three segments (1800 bp each, with 1709 bp; 1621 bp; and 1785 bp effective sequences) for analysis. The nearby segments were designed overlapped to avoid the potential effects of sequence dividing. Six interaction domains with a minimal interaction energy of <− 10 kcal/mol were found in the *IRLnc* and *NR4A3* interaction prediction analysis (Table 2 and Fig. 5). Interestingly, the domain with the lowest minimal interaction energy (− 17.6096 kcal/mol) was located in the 3′UTR region of *NR4A3*. This result indicated that the conserved sequence of *IRLnc* may interact with *NR4A3* mRNA and directly regulate its expression.

**Table 2:** The predicted interaction domain of *IRLnc* and *NR4A3*

Target	Start	Target position	Gene regions	Query	Query position	Energy
*NR4A3*	3331	4198--4236	3’UTR	*IRLnc*	165 -- 202	−17.6096
*NR4A3*	3331	4449--4495	3’UTR	*IRLnc*	139 -- 187	−13.6224
*NR4A3*	1710	3247--3270	Intron	*IRLnc*	148 -- 175	−13.1712
*NR4A3*	1710	2178--2304	Intron	*IRLnc*	77 -- 203	−12.7953
*NR4A3*	3331	3663--3717	3’UTR	*IRLnc*	116 -- 173	−12.1140
*NR4A3*	1710	2931--2971	Intron	*IRLnc*	154 -- 187	−11.7096

Start: the start position of *NR4A3* mRNA sequence. Target position: relative position of the binding sequence on *NR4A3* (5′ to 3′). Query position:relative position of the binding sequence on *IRLnc* conservation domain

### RNA silencing of *IRLnc*

**Fig. 6:**
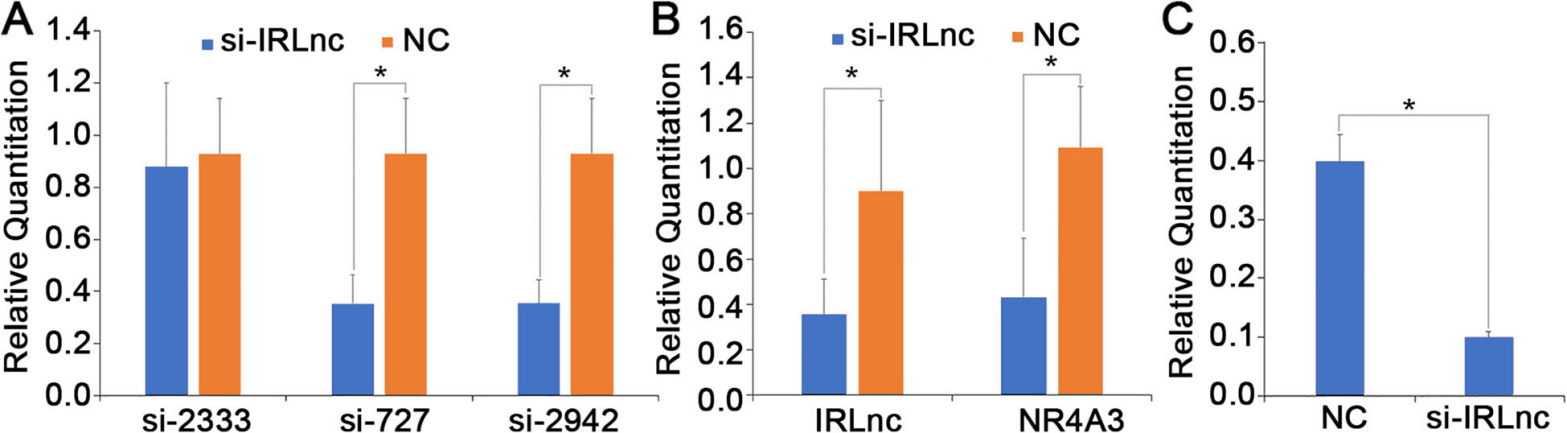
Fold changes of *Sec61B* and *NR4A3*. **a** The transinfected efficiency of three si-IRLnc probes. **b** Expression of *Sec61B* and *NR4A3* in IRLnc silent cells and normal cells. * represent *P*< 0.05, *n*=3 in each group. **c** Differences of NR4A3 protein expression level in normal and RNA-Silencing cells. * represent *P*< 0.05, *n*=3 in each group

Three siRNAs targeting *IRLnc* (si-727, si-2333, and si-2942) were transfected into cells and *IRLnc* interference efficiency was then tested. The results showed that cells transfected with si-727 and si-2942 had significantly different *IRLnc* expression levels than cells transfected with the negative control (NC) siRNA (Fig. 6a, *P* < 0.05), which indicates good knockdown efficiency. Finally, si-727 was selected for subsequent experiments. *IRLnc* silencing significantly decreased the RNA expression of *NR4A3* by approximately 50% (Fig. 6b, *P* < 0.05). Moreover, *IRLnc* silencing significantly decreased the protein expression of NR4A3 (Fig. 6c, *P* < 0.05).

### Co-localization of *IRLnc* and *NR4A3*

**Fig. 7:**
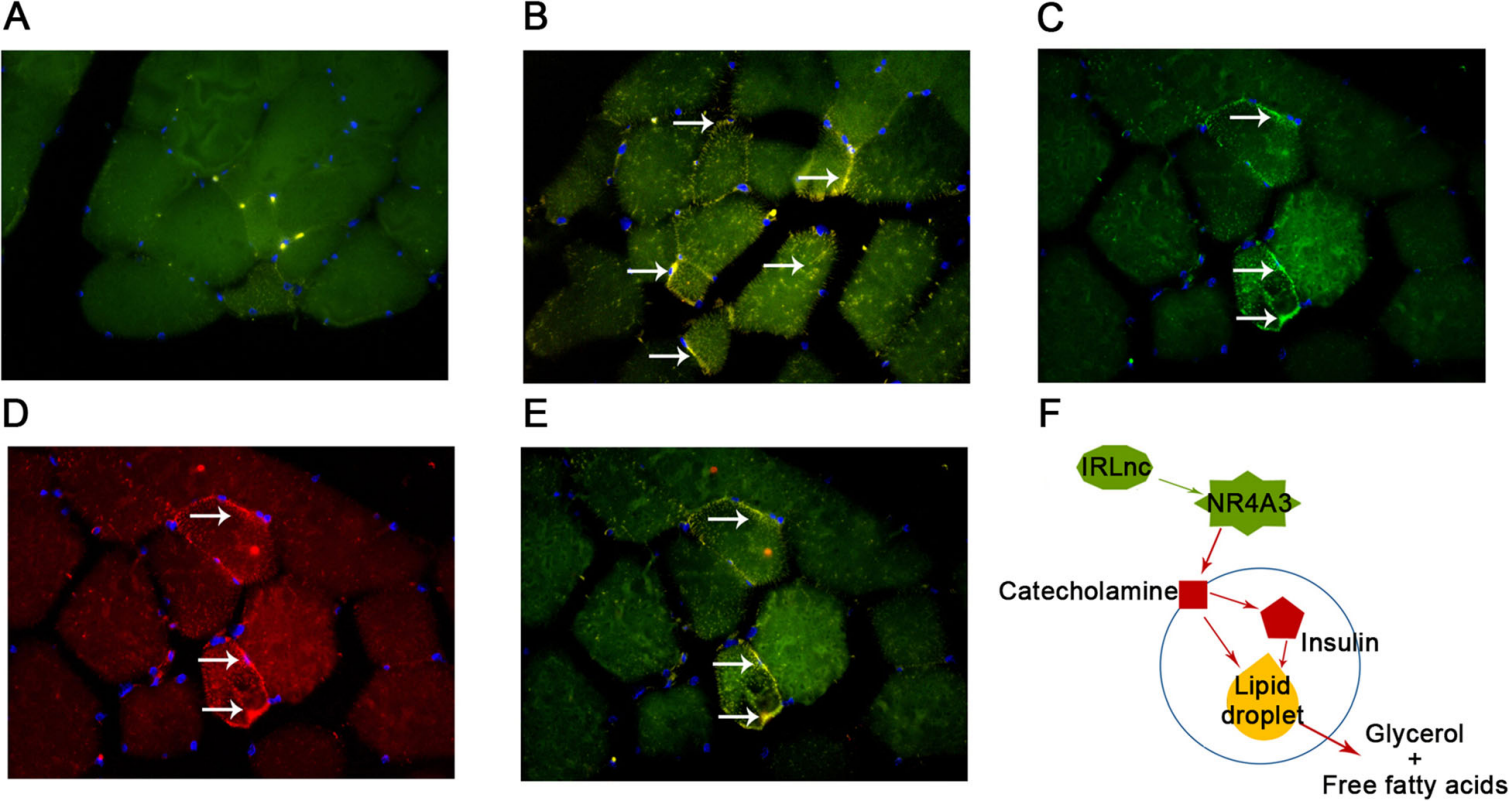
In situ colocalization of *IRLnc* and *NR4A3* mRNA in myoblast and the potential effect pathway of *IRLnc* on fat deposition (*n*=3). **a-e** Representative pseudocolored images of *DapB/NR4A3*, *UBC/NR4A3*, *IRLnc*, *NR4A3*, and *IRLnc/NR4A3* stained with DAPI (nucleus, blue) and for *NR4A3* (red), *DapB* (green), *UBC* (green), and *IRLnc* (green), and the color represent co-expression will be orange (red+green). Total magnification of all images is 20 ×. **f** Potential effect pathway of *IRLnc* on lipolysis. Red line represents inhibit effect; green line represents promote effect

In-situ hybridization analysis showed that, in NCs, there was no co-localization of *IRLnc* and *DapB* (Fig. 7a). However, co-localization of *IRLnc* and *UBC* (Fig. 7b) and *IRLnc* and *NR4A3* (Fig. 7c-e) was observed. Moreover, the results showed that *IRLnc* and *NR4A3* were mainly located at the margin of muscle fibers where intramuscular fat is deposited.

## Discussion

In our research, three pairs of full-sib Large White × Min pigs F2 sows which with extremely different IMF content were selected to do RNA sequencing analysis. Gene annotation and PhastCons analysis were used to mining candidate LincRNAs related to IMF. Co-localization analysis and RNAi-mediated loss of function screens were performed to study the potential interactive mechanism of the candidate LincRNA and its downstream gene.

### Investigation of differential expression transcripts

Min is a native Chinese pig breed with an average IMF of 5.22% and Large White is a European pig breed with an average IMF of 2.00%. The offspring of Large White × Min crossbred (F2) population is an ideal IMF separated model to analyze the genetic mechanism of IMF. We used three pairs of full siblings from the F2 population, so that the genetic background of each pair was consistent. The paired samples model in edgeR was also appropriate for our research design. Although thousands of transcripts and lincRNAs were identified in pig genomes, there were only 26 DE transcripts between the groups with high and low IMF content.

### Function analysis of DE protein-coding genes

Among these 26 transcripts, 15 protein-coding genes were identified and located to known genes. Among the known genes, ten were associated with fatty acid and myocyte formation or metabolism. One of the SNPs in *PPARGC1* (rs8192678[A]) has been reported to be associated with higher triacylglycerol levels (*P* = 0.005) [17]. *FASN* and *LEP* are important factors in fatty acid synthesis [18, 19]. *NR4A3* is a regulator of insulin activity in adipocytes [14, 20]. *ACBD7* could be involved in energy homeostasis and associated to obesity in humans [21]. Specific knockout of *SLC20A*1 could significantly decreased hepatic lipogenesis [22]. A SNP in *PRKAG2* was associated with plasma free fat acid and glycerol measurements [23]. Adiponectin (*ADIPOQ*) has the glucose regulation and fatty acid oxidation function [24]. A SNP in Growth arrest and DNA damage-inducible protein GADD45 Alpha (*GADD45A*) showed significant association patterns for IMF and backfat thickness in Berkshire pigs [25]. *ND6* could participated in regulates mitochondrial fatty acid oxidative metabolism, and two rare variants in *ND6* were associated with BMI [26, 27]. One gene named *LRRC66* was involved in diverse biological processes, including cell adhesion, cellular trafficking, and hormone-receptor interactions [28]. And other genes were mainly associated with diseases such as Mucopolysaccharidosis IIIB [29], stress-induced injury [30], oral-facial-digital syndromes [31], and breast cancer [32]. From the results, intramuscular related genes involved in fat and muscle related function were consistent with previous research, which reported that IMF is regulated through a complex pathway that interacts with muscle, fat, and connective tissue [33, 34].

Our study also identified six DE lincRNAs that may be involved in IMF regulation. These three lincRNAs are located on chromosomes 1, 4 and 10. The lincRNA on chromosome 1 (*IRLnc*) had not previously been reported associated with IMF in pigs. It displayed a 6.48-fold higher level of expression (logFC = 2.69) in pigs with high IMF content compared to those with low IMF content (FDR = 4.80E-12) and as such, was the locus most significantly associated with IMF content. Therefore, we selected *IRLnc* for further research.

### Potential regulatory effect of *IRLnc*

To investigate the potential regulatory effect of *IRLnc*, we first analyzed the *IRLnc* gene and its surrounding genes. Although all of the genes upstream and downstream of *IRLnc* are conserved, *NR4A3* was the only gene adjacent to *IRLnc* that displayed differential expression, according to the results of RNA-seq and RT-qPCR analysis. To confirm these results, we detected the expression of *IRLnc* and *NR4A3* in five kinds of purebred pigs. Laiwu pig is famous breed with extremely high- IMF, Min and Mashen pigs have high IMF, Beijing Black pigs have median IMF, and Large White pigs have median to low IMF. And the expression results in purebred pigs consist with the results in crossbred pigs. This observation implied that *IRLnc* may be a regulatory element for the *NR4A3* gene. Thus, we silenced *IRLnc* in myoblast cells to investigate the influence of *IRLnc* on *NR4A3* RNA and protein expression*.* Silencing *IRLnc* resulted in a decrease in *NR4A3* expression, thus confirming the speculation that *IRLnc* may regulate *NR4A3*.

The results of RNA-RNA interaction prediction were also positive. We found that the 292-bp conserved sequence of *IRLnc* had a very low energy requirement to interact with the 3′UTR region of *NR4A3* mRNA. Therefore, we inferred that *IRLnc* may regulate the expression of *NR4A3* by binding to its regulatory domain. The finding that *IRLnc* and *NR4A3* are co-localized also supports our hypothesis. Together, these results implied that *IRLnc* may affect *NR4A3* expression by directly binding to its regulatory domain.

In previous research, the NR4A family, especially *NR4A2* and *NR4A3*, have been shown to be unnecessary for adipogenesis [35]. Thus, we inferred that NR4A3 may not affect fat deposition through the adipogenesis pathway. Previous studies have shown that *NR4A3* gene expression is reduced in skeletal muscles and adipose tissues from multiple rodent models of insulin resistance [14, 20]. Insulin sensitivity is known to be closely related to fat deposition. In the research of Walton et al. (2013), over-expression of *NR4A3* induced a decrease in the concentration of circulating catecholamines, leading to poor insulin sensitivity and increased low-density lipoprotein levels [36]. Additionally, poor insulin resistance leads to a decrease in lipolysis [37]. In summary, we propose that *IRLnc* directly regulates the expression of *NR4A3*, which then regulates catecholamine catabolism and finally, IMF deposition, by regulating insulin sensitivity (Fig. 7f). A recent study showed that IMF directly modulates muscle insulin sensitivity [38] and we propose that this process may occur through the same pathway. However, the expression regulatory activity of *IRLnc* and the pathway mediating the effect of *IRLnc* on IMF require further investigation.

## Conclusions

This study performed RNA sequencing analysis using an IMF character segregation population, and provided 26 DE transcripts in high- and low- IMF pigs. This research also provided a global view of the complexity of the mRNA and lincRNA transcriptome in pigs with different IMF content. Using bioinformatic analysis, co-localization analysis and RNAi-mediated loss of function screens, we also explored the potential interactive mechanism of the candidate LincRNA (*IRLnc*) and its downstream gene (*NR4A3*). *IRLnc* that may influence IMF decomposition maybe a potential marker for meat quality selection. Furthermore, as *IRLnc* could directly regulate the expression of *NR4A3* which associated with insulin resistance, we inferred that *IRLnc* may be a potential therapeutic target for insulin resistance and type 2 diabetes.

## Methods

### Animals and sample collection

All of the animals used in our research were obtained from the experiment pig farm of Institute of Animal Science, Chinese Academy of Agricultural Sciences (CAAS). Before slaughter, all of the pigs are stunned using 80%-concentrate carbon dioxide for 45 s. When the pigs were confirmed stunned, they were then hoisted on a rail and exsanguinated via carotid artery and the jugular vein. After the blood is gone, pigs were handled on a Stork slaughter process line (Stork B. V, Naarden, Dunth) with standard procedure (removal of hair, eviscerated, cut into two-halves, and so on). In this study, a total number of 46 pigs were used to collect tissues samples, and at least 3 pigs in each group (mostly 5 pigs, as described in each part below) were select to meet calculation power. After experimentation, all of the pigs were sold to the slaughter house.

Six F_2_ sows (three pairs of full siblings) from a Large White × Min resource population (678 pigs, including 602 F2 individuals, all of the pigs were fed in same diet and raised in same condition. The tissue sampling condition are also uniformed) were used in this study for RNA extraction. In each pair of siblings, there was one high-IMF pig and one low-IMF pig (Table 3). IMF content was measured using an ether extraction method (Soxtec Avanti 2055 Fat Extraction System; Foss Tecator, Hillerød, Denmark).

**Table 3:** Phenotype and pedigree information of three full-sibling pigs

ID	Group	Group ID	Father ID	Mather ID	IMF content (%)
19,803	Low	L1	721,205	723,604	0.9
1,015,105	Low	L2	706,601	706,204	1.41
1,119,609	Low	L3	700,105	709,602	1.08
19,809	High	H1	721,205	723,604	5.56
1,015,103	High	H2	706,601	706,204	5.94
1,119,605	High	H3	700,105	709,602	7.51

*IMF* intramuscular fat

### RNA isolation, sequencing, and mapping

Total RNA was extracted and purified using TRIzol (Invitrogen, Carlsbad, CA, USA) and a RNeasy Mini Kit (Qiagen, Hilden, Germany). A total of 3 μg of RNA per sample was then used for RNA sample preparation. After amplification, PCR products were purified (AMPure XP system; Beckman Coulter, Brea, CA, USA) and library quality was assessed on a Bioanalyzer 2100 system (Agilent Technologies, Santa Clara, CA, USA). Each library was sequenced on a HiSeq 2000 platform (Illumina, San Diego, CA, USA) at the Novogene Bioinformatics Technology Cooperation (Beijing, China). Raw data in fastq format were firstly filtered to clean paired-end data using in-house Perl scripts. A gene model annotation system (Ensembl version 92), the reference genome (*Sus scrofa* 11.1), and associated files were directly downloaded from the Ensembl website (ftp://ftp.ensembl.org/pub/release-92/fasta/sus_scrofa/dna). An index of the reference genome was constructed using the Bowtie v2.0.6 package [39], with default parameters, and clean reads were aligned to the reference genome using TopHat v2.0.9 [40], with default parameters.

### Identification of transcript units

Cufflinks v2.0.2 [41] was used to assemble the aligned reads for each sample. Cuffcompare v2.0.2 [42] was then used to generate intergenic transcripts for each sample assembly. To acquire high-confidence transcripts, two criteria were used to filter the transcripts using in-house Perl scripts: (1) RNA-seq reads must have covered more than 80% of predicted exon nucleotides for a transcript, (2) in at least one sample, there must have been more than three clean reads mapping to the predicted splice structure. Finally, fragments per kilobase of exon, per million fragments mapped values were obtained using Tophat v2.0.9 with --no-novel-juncs and Cufflinks v2.0.2 with -G.

### Differential expression analysis

The identification of differentially expressed (DE) transcripts between low-IMF and high-IMF pigs was performed in edgeR using a paired samples model [16]. Significant DE transcripts were determined with the following criteria: false discovery rate (FDR) < 0.1 and log2 fold-change (logFC) value more than 0.58 (log2[1.5, 2]) or less than − 0.58 (log2[0.67, 2]).

### Reverse transcription quantitative polymerase chain reaction validation of DE genes

cDNA was synthesized from total RNA using a PrimeScript reverse transcription (RT) reagent kit (Takara Bio Inc., Kusatsu, Japan). Primers were designed for all 26 DE transcripts using Primer 6 software (Table S1). cDNA samples from 10 pigs (five with high IMF content and five with low IMF content) were used as templates for quantitative polymerase chain reaction (qPCR). Reactions were performed on an ABI 7900HT Real-Time System (Applied Biosystems, Foster City, CA, USA) with a 15-μL mixture consisted of 1.5 μL of cDNA, 150 nM of each of the forward and reverse primers, and SYBR® Green PCR Mixture (ABI part number 4472908). Standard PCR cycling conditions were used. The glyceraldehyde-3-phosphate dehydrogenase (*GAPDH*) gene was used as a control and relative gene expression levels were calculated using the 2^−ΔΔCt^ method [43], where the delta cycle threshold (ΔCt) is the Ct of target gene minus the Ct of *GAPDH* and ΔΔCt is the ΔCt of the target gene minus the average ΔCt of all individuals. A Student’s t-test was used to analyze the differences in expression between the low- and high-IMF groups.

### Identification of sequence homology with 99 vertebrates

NCBI BLASTn was used to identify the sequence homology of selected lincRNAs with 99 vertebrates, including humans. All sequences were retrieved from the University of California Santa Cruz database and the PhastCon conservation plot and consensus logo plot were drawn using in-house R scripts [44].

### Expression of IMF-related lincRNA and its upstream and downstream genes

The pigs described above (five low-IMF content and five high IMF content) were selected to detect differences in the expression of IMF-related lincRNA (*IRLnc*), translocon beta subunit (*Sec61B*), and nuclear receptor subfamily 4 group A member 3 (*NR4A3*) using RT-qPCR. Primers for these three transcripts were designed using Primer 6 software (Table S1). Standard PCR cycling conditions were used. The *GAPDH* gene was used as control and relative gene expression levels were calculated using the 2^−ΔΔCt^ method. A Student’s t-test was used to analyze the differences in expression between the low- and high-IMF groups. Five pigs in each breed (Laiwu, Min, Mashen, Beijing black and Large White) were also selected to detect differences in the expression of *IRLnc*, *Sec61B*, and *NR4A3* using RT-qPCR. The statistical methods were same as that used in IMF different pigs.

### LincRNA-RNA interaction prediction

LincRNA-RNA interactions between the 292-bp conserved sequence of *IRLnc* and *NR4A3* mRNA were predicted using IntaRNA software (server version 4.4.2) [45, 46], with the following parameters: number of (sub) optimal interactions = 5 and min. Number of basepairs in seed = 7.

### Cell isolation, tissue culture, and siRNA knockdown of *IRLnc*

Longissimus dorsi muscle tissues were obtained from one newborn pig. After fractionation and collagenase digestion (12,500 U I/II collagenase/kg of tissue; Sigma, St Louis, MO, USA), primary myoblasts, enriched in bottom stromal vascular fractions, were resuspended and cultured to confluence in Dulbecco’s modified Eagle’s medium supplemented with 10% (vol/vol) fetal bovine serum. After 2 d, cells were incubated in culture medium containing insulin (5 μg/mL) for another 2 d and the culture solution was then changed every 2d.

Three IRLnc-specific siRNAs and one NC siRNA were designed by GenePharma (Shanghai, China; Table S2). When cultured primary myoblasts reached 70–80% confluence, siRNAs (150 nM) were transfected using DharmaFect2 (5 μL/mL), according to the manufacturer’s instructions (Dharmacon, Lafayette, CO, USA). Twelve hours after transfection, cells were induced to differentiate and were then harvested for downstream RNA and protein analysis on the fourth day after differentiation. Total RNA and protein were isolated from the cells using standard procedures. The differences in *Sec61B* and *NR4A3* gene expression between normal and *IRLnc*-knockdown cells were detected using RT-qPCR. Moreover, the differences in NR4A3 protein between normal and *IRLnc*-knockdown cells were detected using western blotting, and the signals were normalized to β-actin expression.

### In-situ hybridization co-localization of *IRLnc* and *NR4A3*

The longissimus dorsi muscles of three 0-d-old piglets were collected for in situ hybridization co-localization analysis. Tissues were freshly harvested, immediately fixed in 4% formalin, processed for paraffin embedding using standard protocols, and then sectioned onto SuperFrost Plus slides (Fisher Scientific, Waltham, MA, USA) at thickness of 5 mm.

Fluorescent probes were designed using *IRLnc*, *NR4A3*, *UBC*, and *DapB* sequences and synthesized by Advanced Cell Diagnostics (Newark, CA, USA). The *NR4A3* probe used the C2 channel (red) and other probes used the C1 channel (green). RNA in-situ hybridization experiments were performed using the Multiplex Fluorescent Reagent Kit V2 kit (Advanced Cell Diagnostics), according to the manufacturer’s protocol. *DapB* was used as the NC and *UBC* as the positive control. Fluorescent images were acquired using a TCS SP8 confocal microscope (Leica, Wetzlar, Germany) and *IRLnc/NR4A3*, *IRLnc/UBC*, and *DapB/NR4A3* co-localization was analyzed using Leica Application Suite X v3.1 software.

### Statistical analysis

The data obtained are expressed as mean ± SE, a t-test was used to evaluate the statistical significance of the 2-part comparisons of expression difference. Three replicates in each group were used in vitro. Statistical analysis was carried out using SAS 9.4 statistical software, and the statistical significance was set at *P*< 0.05.

## Supplementary Information

**Additional file 1: Table S1.** cn- stream genes. **Table S2.** Primers of DE si-RNA of *IRLnc.*

## Data Availability

The sequencing data used in the current study have been submitted to the Genome Sequence Archive, with the accession number CRA001645.

## Abbreviations

ΔΔCt: Delta delta cycle threshold
DE: Differential expression
DMEM: Dulbecco’s Modified Eagle’s medium
FDR: False discovery rate
*GAPDH*: Glyceraldehyde-3-phosphate dehydrogenase
GSA: Genome sequence archive
GWAS: Genome-wide association study
IMF: Intramuscular fat
NC: Negative control
RT-qPCR: Reverse transcription quantitative polymerase chain reaction
QTLs: Quantitative trait loci
Sec61B: Translocon beta subunit
SNPs: Single nucleotide polymorphisms
UCSC: University of California Santa Cruz
CPM: Counts per million
miRNAs: microRNAs
*NAGLU*: N-Acetyl-Alpha-Glucosaminidase
*Novel*: Novel gene
*ACBD7*: Acyl-CoA Binding Domain Containing 7
*PPARGC1*: Peroxisome proliferator-activated receptor-gamma coactivator-1
*IRLnc*: IMF-related LincRNA
*GADD45A*: Growth Arrest And DNA Damage-Inducible Protein GADD45 Alpha
*NR4A3*: Nuclear receptor subfamily 4 group A member 3
*SRXN1*: Sulfiredoxin 1
snRNAs: Small nuclear RNAs
rRNAs: Mitochondrial ribosomal RNAs
*LEP*: Leptin
*SLC20A1*: Solute Carrier Family 2 Member 1
*FASN*: Fatty acid synthase
*PRKAG2*: Protein Kinase AMP-Activated Non-Catalytic Subunit Gamma 2
*ND6*: Mitochondrially Encoded NADH: Ubiquinone Oxidoreductase Core Subunit 6
*ADIPOQ*: Adiponectin, *SUSD3*: Sushi Domain Containing 3
*C2CD3*: C2 Calcium Dependent Domain Containing 3
*LRRC66*: Leucine Rich Repeat Containing 66
