# Supplementary material for: IRLnc: a novel functional noncoding RNA contributes to intramuscular fat deposition
Source: BMC Genomics. 2021 Feb 1;22:95. doi: 10.1186/s12864-020-07349-5 (PMC7849149; doi:10.1186/s12864-020-07349-5)
Supplement: Supplementary file 1 — Additional file 1: Table S1. cn- stream genes. Table S2. Primers of DE si-RNA of IRLnc. [file 12864_2020_7349_MOESM1_ESM.docx]

**Table S1 Primers of DE transcript an up-and down- stream genes**

| Genes | Sense strands (5’-3’) | Anti-sense strands (5’ -3’) |
| --- | --- | --- |
| *IRLnc* | AAGGTCAGTGGTGGATAGC | ATGTTGATTTCCTATGCCAGAG |
| *ACBD7* | TGAGAACCAGTAACAAGACC | ACCACAGAAGGAGGAAGG |
| *FASN* | TCGGAGAACCTGGAGGAGTT | TGTTCGCCTGCTTGGAGTG |
| *NR4A3* | CTCAAGCCTTCCTGCCTGTA | CGTGGTGGTGATGGTGGTA |
| *SRXN1* | GGTGGCTGATGGTATTGG | CGACTCCGTTCTTCCTTG |
| *LEP* | CAAGACCATAACAGCAGACT | CTCCAGGCAATCCACTTC |
| *ND6* | TATACTACTGCTATGGCTACTG | TCTACTTCCTCTTCCTTCAAC |
| *Novel3* | CTCCACCATCTGCTGTTG | CTGAGGCTCCTGGTAAGT |
| *C2CD3* | CTGAGTTAGGTGGTTGGAA | GCTGAATGACGAGAGTAGT |
| *Sec61B* | GTTCCAGTATTGGTTATGAGTCTTC | TTCCTTCCTTCTTCAGATGACAG |

*Note:* *IRLnc*: IMF-related LincRNA, *ACBD7*: Acyl-CoA Binding Domain Containing 7, *FASN*: Fatty acid synthase, *NR4A3*: Nuclear receptor subfamily 4 group A member 3, *SRXN1*: Sulfiredoxin 1, *LEP*: Leptin, *ND6*: Mitochondrially Encoded NADH: Ubiquinone Oxidoreductase Core Subunit 6, *Novel3*: novel gene 3, *C2CD3*: C2 Calcium Dependent Domain Containing 3, *Sec61B:* Sec61 translocon beta subunit.

**Table S2 Primers of siRNA probes**

| Genes | Sense strands (5’-3’) | Anti-sense strands (5’ -3’) |
| --- | --- | --- |
| siRNA-sus-727 | GGAGUUGUCAUAUGAUGAATT | UUCAUCAUAUGACAACUCCTT |
| siRNA-sus-2333 | GCUUCAGGCUGCAUGGAAUTT | AUUCCAUGCAGCCUGAAGCTT |
| siRNA-sus-2942 | GCUUCAUAAGAGGAACUAATT | UUAGUUCCUCUUAUGAAGCTT |
